# Plant-Based Burgers with Reduced Texture Additives: A Comparative Study of Methylcellulose and Sodium Alginate

**DOI:** 10.3390/foods14081373

**Published:** 2025-04-16

**Authors:** Irene Peñaranda, María Belén López Morales, María Dolores Garrido, Macarena Egea

**Affiliations:** Department of Food Science and Technology, Veterinary Faculty, University of Murcia, Espinardo, 30100 Murcia, Spain; irene.penaranda@um.es (I.P.); mbelen@um.es (M.B.L.M.); mgarrido@um.es (M.D.G.)

**Keywords:** soybean, methylcellulose, sodium alginate, plant-based burger, texture, reduced additives, sensory

## Abstract

The limited number of additives in plant-based burgers is related to clean label consumer perception, which influences purchase intention. Starch is typically combined with other texturing agents to replicate the texture and mouthfeel of meat burgers. It is necessary to reformulate these products following consumers’ trends, who prefer healthier products with fewer additives. Two hydrocolloids with significant commercial application and different functionality were evaluated: methylcellulose (M) or sodium alginate (SA). Four formulations were developed, two containing starch (M+S and SA+S) and two without starch (M and SA). The alginate burgers provided samples with high water retention capacity and a cohesive and adhesive texture, superior to the samples with methylcellulose, without the need to add starch, due to their stabilizing, thickening, and gelling properties derived from their “egg-crate” structure when gelled. Furthermore, sensory analysis indicated that the sodium alginate burgers had a softer and creamier texture. In contrast, starch removal in the methylcellulose burgers enhanced their appearance due to gel transparency and desirable textural properties, akin to those of meat. These results promote using a 3 g/100 g methylcellulose solution as the sole binding agent in soybean burgers to achieve a product with reduced additives.

## 1. Introduction

Meat is one of the main components of the human diet, whose nutritional purpose is to supply high biological value protein (18–20%) and other essential micronutrients such as minerals and vitamins [1]. Considering that the world population will increase by more than 2 billion people in the next 20 years, the demand for meat consumption is expected to increase by 50% by 2050 [2], making it necessary to consider a change in the way food is produced, towards more sustainable systems with less environmental impact and greater resilience.

Food production is one of the activities with the greatest environmental impact, considering that it is responsible for 30% of greenhouse gas emissions and the consumption of large amounts of water, which has an impact on the global water footprint, as well as the use of energy and other natural resources [3]. These immense environmental and socio-economic problems, such as deforestation, food security, environmental pollution, animal welfare, etc., have raised awareness among consumers, so that they are increasingly searching for sustainable and environmentally friendly food, which motivates them to adopt vegan, vegetarian, or flexitarian diets or to simply want to reduce their meat consumption [4].

These dietary changes have prompted a new way of producing protein-rich foods from plant sources as an alternative to animal protein, with the main source of plant protein being texturized soybean [5], due to its functional properties, such as water retention, emulsification, and fat absorption capacity [6]. Hence, the meat analogue sector is gaining popularity as a healthier and more sustainable alternative, and it is also one of the most innovative and cutting-edge food sectors in the development of new products [7]. The current supply of plant-based burgers is increasing, due to their convenience as a ready-to-eat product, especially in areas where they traditionally have high burger consumption, such as the USA, Latin America, or European countries, but also in expansion markets as Asia [3].

However, it must be considered that the creation of plant-based meat analogues that meet the needs of consumers is a major challenge. While the nutritional aspects of meat analogues are essential, the sensory and textural properties also play a crucial role in consumer acceptance. These products must not only mimic the nutritional profile of meat but also the appearance and texture, especially the mouthfeel, as it is very important for good marketing [7]. For this reason, numerous efforts have been made to combine different ingredients and culinary techniques to achieve an analogue structure that resembles that of animal meat.

However, despite many innovations, manufacturers have not been able to meet the current demands of consumers, who associate fewer additives with more natural and safer products, aligning with the growing demand for transparency and clean labels [8]. The formulation of meat analogues requires a large amount of ingredients and/or additives, apart from the vegetable protein source, that act as binders, such as starch, maltodextrin, methylcellulose, alginates, etc., to improve the cohesion and stability of all ingredients through protein, water, and lipid interactions in the system [9,10]. Typically, the use of starch combined with several hydrocolloids is incorporated in this type of product to achieve suitable textural properties that are more like meat, with the main binder used being starch [11]. Birke Rune et al. [12] concluded that the length of the ingredient list is more important for consumer perception of a clean label in plant-based burgers than chemical additives per se. Starch is a plant energy-reserve polysaccharide that is widely used in large quantities in meat analogues as a texture-modifying agent, due to its low cost in its refined form [13], but with a low acceptance from consumer concerned about the effect on health due to its rapid absorption, which increases the glycaemic index, and its high caloric intake [14]. It is therefore a challenge for the meat analogue sector to create formulations without starch and limiting hydrocolloid combinations with good textural and sensory properties that contribute to satisfying consumer demands [15].

Methylcellulose (M) and sodium alginate (SA) are the main hydrocolloids with the highest volume of commercial use included in the IMR International (Hydrocolloid Information Center) after starches and gelatine [11]. Methylcellulose is a synthetic cellulose polymer produced by substituting methyl groups (-CH3) from the glucose units of natural cellulose. This chemical modification grants it a high functionality to gel, by forming fibrils when heated, resulting in thermoreversible, highly heat-resistant, firm, and transparent gels that require heat to be functional [16,17]. When consumed, it increases fullness and satiety perceptions due to improved and slowed down gastric emptying and increased gastric distension [18]. On the other hand, sodium alginate, at the molecular level, is a seaweed-derived natural hydrocolloid that does not require heat to be functional. As it is a sodium salt of alginic acid, composed of D-mannuronic and L-guluronic acid units, it forms viscous solutions in the presence of water [19]. Its functionality is affected by the presence of divalent ions, such as calcium, which provides thermally irreversible, cohesive, and viscous ionic gels [20]. In addition, this additive can regulate food intake and glycaemia by enhancing gastric distension and delaying gastric emptying [21].

Based on the above, the present study aimed to evaluate the effect of starch elimination as a binding agent on the textural and sensory properties of textured soybean burgers elaborated with a unique hydrocolloid.

## 2. Materials and Methods

### 2.1. Raw Materials

Texturized soybean protein (51.0 protein, 15.0 dietary fibre, 1.3 lipids, and 20.0 carbohydrates g/100 g), spices, and oil (Hacendado) were from Valencia, Spain. The corn starch (Maizena) and sodium alginate (E-401, Tradissimo) were from Barcelona, Spain. Methylcellulose (E-461, Natural de mezclas) was from Murcia, Spain.

#### Preparation of Plant-Based Burgers

The burgers were prepared according to the method described by Botella-Martínez, Viuda-Martos, Fernández-López, Pérez-Alvarez, and Fernández-López [22] and by Peñaranda, Garrido, García-Segovia, Martínez-Monzó, and Igual [23] with slight modifications. Four different formulations were prepared: two to mimic commercial formulations where starch is used (controls), adding methylcellulose and sodium alginate, respectively (M+S and SA+S), and two without starch (M and SA) to study the use of this unique additive. These plant-based burgers were prepared in the pilot plant of the Research Center (CIAVYS-VITALYS, University of Murcia), using the formula from Table 1. For the preparation of the methylcellulose solution, it was first conditioned at 3 g/100 g, hydrated at 4 °C for 24 h for better dispersion, and dissolved at 55–60 °C. In the 7 g/100 g sodium alginate solution, a 5 g/100 g calcium chloride solution was prepared, and 2.5 mL of it was added to the 7 g/100 g alginate solution and dissolved at 55–60 °C. Once both solutions were conditioned, the remaining ingredients were weighed. Soybean protein was hydrated for 20 min at 23 °C. Then, it was crushed in a grinder (Moulinex DP805GBP, Group SEB, Alençon, France) and added to the methylcellulose or sodium alginate, after which the first homogenization was carried out. Next, the starch for the formulations containing starch (M+S and SA+S), the spice mixture, and the olive oil were added and mixed in a homogenizer (Thermomix TM 31, Vorwerk, Wuppertal, Germany) until a homogeneous mass was obtained. The mixtures of the four formulations were then manually shaped into 40 g burgers. All the samples were produced in triplicate. Three batches of each formulation were elaborated on different days following the same process.

### 2.2. Physicochemical Analyses

**Figure 1:**
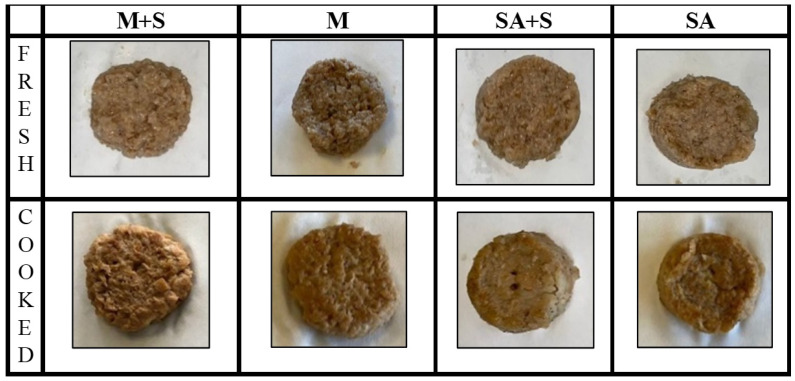
Appearance of fresh and cooked plant-based burgers with different hydrocolloids. M+S (burgers made with methylcellulose as a binder with starch), M (burgers made with methylcellulose as a binder without starch), SA+S (burgers made with sodium alginate as a binder with starch), and SA (burgers made with sodium alginate as a binder without starch).

Physicochemical analyses were carried out on fresh (colour and water holding capacity) and cooked (cooking loss and mechanical properties) samples (Figure 1). Images of the samples were taken using a mobile phone camera without automatic correction, with a neutral white background (paper filter), utilizing natural light and the room’s artificial lighting, positioning the camera at a height of 20 cm.

The colour was determined using a CR-400 Chroma Meter (Minolta Ltd., Milton Keynes, UK) calibrated against a standard white tile (8 mm diameter aperture, d/0 illumination system, D65 illuminant, and a 2° standard observer angle), on the sample surface from three randomly chosen spots [23]. The following CIELAB colour coordinates were obtained: lightness (L*), redness (a*), and yellowness (b*). The psychophysical variables °hue (h*) and chroma (C*) were calculated from the colour coordinates by using the following equations:

Chroma = (a*2 + b*2)1/2(1)

Hue = tan − 1(b*/a*)(2)

The water holding capacity (WHC) was determined using the method developed by Grau and Hamm [24]. This involves applying a pressure of 1 kg to a sample and measuring the amount of water released from a 0.3 g sample placed on a Whatman No. 540 filter paper and pressed for 10 min. The % WHC was calculated using the following formula:

% released water = [(W*f* − W*i*)/ W*s*] × 100 (3)

where:

W*f* = final weight of the paper (g);

W*i* = initial weight of the paper (g);

W*s* = sample weight (g);

WHC (%) = 100 − % released.

To determine the cooking loss (CL), the weight difference method described by Wi et al. [25] was used. The meat analogue samples were cooked at a temperature of 180 °C on a Velox CG-1S griddle (Silesia, Spain, Barcelona) for 10 min, until an internal temperature of 80 °C was reached, as measured by a penetration probe. After cooking, the samples were allowed to cool for 30 min to reach room temperature. The CL was calculated by comparing the weight of the samples before and after cooking and expressed as a percentage using the following formula:

CL (%) = (W1 − W2)/W1 × 100(4)

where:

W1: weight of uncooked plant-based burger (g);

W2: weight of cooked plant-based burger (g).

A texture profile analysis (TPA) of the plant-based burgers was performed using a CT310K Texturometer from Brookfield CNS Engineering Labs. Inc., Harlow, UK, and TexturePro CT V1.8 software, following the protocols outlined by Lee and Hong [26] and Peñaranda et al. [23]. The samples were prepared by cutting them into 2 cm diameter cylinders (23 °C). The samples were then subjected to a double cycle compression test with a cylindrical probe (TA 10, 10 mm diameter) and a 25 kg load cell, compressing them by up to 50% of their original height. The force-time deformation curves were obtained with a speed of 2.5 mm/s and a trigger point of 5 g. The mechanical properties measured were hardness (g), adhesiveness (mJ), chewiness (mJ), cohesiveness, elasticity (mm), and resilience (J/m^3^). Each sample was replicated six times.

### 2.3. Sensory Analysis

The sensory analysis was carried out by 6 trained panellists (4 women and 2 men) from the Food Science and Technology group at the University of Murcia (Spain). They were selected based on their prior experience in sensory evaluation of meat substitutes [23]. The sensory evaluation study followed the recommendations of the Declaration of Helsinki and strictly adhered to the guidelines of the research ethics committee of the University of Murcia for the sensory analysis of food with trained panels. Participants gave their informed consent through the statement “I am aware that my answers are confidential and I agree to participate in this study as a trained panellist” where an affirmative answer was required to enter the tasting panel. Before proceeding with the sensory study, the previous experience of the panel was evaluated, after which retraining and validation took place [27]. The panellists underwent five 1.5 h theoretical and practical sessions to become familiar with plant-based burgers and to identify the relevant descriptors and their corresponding ranges (Table 2).

To evaluate the plant-based burgers’ attribute intensity, a quantitative descriptive analysis (QDA) test was conducted using a 10-point unstructured scale (0: not perceptible; 10: very intense) [28]. Samples were cooked on a Velox CG-1S griddle (Silesia, Barcelona, Spain) at 180 °C for 12 min, until the internal temperature reached 80 °C (measured using a T200 portable thermometer from Digitron Instrumentation Ltd., Hertford, UK). The cooked samples were cut into 2 × 2 cm pieces, wrapped in coded aluminium foil, and kept in a sand bath at 60 °C until tasting [29]. The order of sample presentation was randomized and balanced to account for order and carryover effects [30]). The analyses were carried out in the morning at 10:30 h. in a standardized sensory room [31] at the Food Science and Technology Department at the University of Murcia. Each panellist evaluated three samples from each creation (4 formulations x 3 replicates), in a total of six sessions. Mineral water and unsalted breadsticks were provided for mouth rinsing between samples.

### 2.4. Statistical Analysis

All data were analysed using the SPSS 28 statistical package (SPSS, Chicago, IL, USA). Data for colour (L*, a*, b*, chroma and ºhue), water holding capacity (WHC), cooking loss (CL), and mechanical properties were analysed using a two-way analysis of variance (ANOVA), considering the effects of starch’s presence in the formulation (S: starch-containing) and the hydrocolloid we used (M: methylcellulose and SA: sodium alginate) as fixed sources of variation and manufactured batches as a random effect. For the sensory analysis data, a two-way ANOVA was performed; the sensory attributes were considered the dependent variable, and manufactured batches, panellists, and sessions were adjusted as random effects. Pearson’s correlation coefficient was calculated for the physical, chemical, and sensory variables. All tests were conducted at an α = 0.05 significance level.

## 3. Results and Discussion

### 3.1. Physicochemical Analyses

Table 3 shows the results of the colour, water holding capacity, and cooking losses of the different plant-based burger formulations (M+S, M, SA+S, and SA). For the CIELab colour, significant differences were observed in the effect of the hydrocolloid (methylcellulose and sodium alginate) in all coordinates (*p* ≤ 0.05), except for lightness (L*) and °hue (h*) (*p* > 0.05). All the samples obtained average values of L* in the 58-to-63 range, and h* obtained values between 62 and 64, indicating that the samples were light-coloured [32]. Concerning the coordinates a*, b*, and C*, the samples with methyl cellulose (M and M+S) had the most reddish (a*) and yellowish (b*) colouring with the highest saturation (*p* ≤ 0.05). In the work of Bakhsh et al. [16], on the effect of hydrocolloids on different plant proteins, including methylcellulose, they observed similar results to those from our study in plant-based burgers made with non-starch methylcellulose.

The methylcellulose gel has a transparent colouring that may not have interfered with the colour of textured soybeans, while the calcium alginate gel has a higher opacity due to the formation of calcium complexes, which prevent the passage of light. Since alginate is a naturally occurring ionic polysaccharide, it could form hydrogels when divalent cations such as Ca^2+^ are added [33].

In addition, statistically significant differences were obtained in brightness with both hydrocolloids, as well as in the b* and C* coordinates in the burgers made with methylcellulose (*p* ≤ 0.05). This shows how the lightness of the plant-based burger is reduced with the elimination of starch (*p* > 0.05), as starch is characterised by a white colour, with a lightness close to 97 out of 100 [34]. Furthermore, it has been shown that L* is affected by the water holding capacity and free water on the surface of the food. This is because bubbles are introduced in the free water during the cutting of the dough, which will produce a higher reflection of light, thus increasing this parameter [35]. In contrast, the non-starch samples prepared with methyl cellulose and sodium alginate obtained the highest b* and C* values (*p* ≤ 0.05). Starch forms white and opaque gels that inhibit the yellowing of the soybean [34], providing the samples with lower b* coordinate values. Concerning chroma (C*), the work by Zahari et al. [35] discussed how the effect of C* is linked to the effect of the a* and b* coordinates, with the same factors modifying both, as it occurred in our results with the b* coordinate.

For the water retention capacity (WHC), a significant effect of the type of hydrocolloid was observed (*p* ≤ 0.05), with the plant-based burgers made with alginate having a higher water retention capacity than those made with methylcellulose. Although differences between the hydrocolloids were observed, these were not very pronounced, with all the burgers obtaining high WHC values of around 84.05–90.25%. Similar results were presented by Zhou et al. [36] in plant protein burgers with a WHC of 94 ± 4%. WHC is one of the fundamental properties of meat analogues, as it has a great influence on the yield and sensory acceptability of the product, and it is therefore of great importance in our meat analogue [25]. According to Yao et al. [37], WHC measures the ability of the protein to retain water, which is affected by protein–polysaccharide interactions that are based on electrostatic forces, hydrogen bonds, and the microstructure of the hydrocolloid. Therefore, alginate has a greater capacity to retain water inside, due to its “egg-crate” structure, compared to methylcellulose. In the work by Lee and Hong [7] on soy burgers, the authors also observed the high water retention properties of alginate.

In contrast, no effects of starch elimination in the formulation of the plant-based burgers were observed for the WHC (*p* > 0.05). The use of high concentrations of methylcellulose (3%) and the three-dimensional alginate matrix were sufficient to reduce the amount of water released [23,37] in each treatment (M and SA), respectively, without the need for starch addition.

For cooking losses, significant differences were observed in the effect of hydrocolloids on burgers with starch (*p* ≤ 0.05) and for the effect of starch on burgers with sodium alginate (*p* ≤ 0.05). Burgers with alginate had the lowest water losses after cooking, in agreement with the results obtained for the WHC, as these parameters are inversely related [25]. As previously mentioned, this is possibly due to the structure of alginate, providing a consistency that prevents the diffusion of water during cooking [38].

At the same time, an effect of the addition of starch was observed in the plant-based burgers made with alginate (*p* ≤ 0.05), with higher losses in the absence of starch. During cooking, certain reactions take place, such as protein aggregation and denaturation, and the evaporation or diffusion of water, which affect the emulsification capacity of the burger dough and are therefore responsible for weight losses during cooking, mainly caused by water loss [22]. It is therefore very important that the nature of the protein is taken into consideration, as it has been observed that starch has a low thermal resistance [13], so that a well-structured protein network is necessary to favour the swelling of the starch granules to prevents the leakage of solids or liquids during cooking [39]. In general, losses were similar to those from other studies, where the authors observed how the use of high concentrations of texturized protein ingredients and a mixture of binding agents during the preparation of burgers, such as gums, gluten, starch, etc., prevented the loss of solids or liquids during cooking, providing a loss value of around 10–15% [16,36,40].

Table 4 shows the mechanical properties of the plant-based burgers, due to the effect of both hydrocolloids (methylcellulose and alginate) and the elimination of starch, with no statistically significant differences observed for any of the parameters analysed (*p* > 0.05), except for adhesiveness, resilience, and cohesiveness (*p* ≤ 0.05). Adhesiveness was only affected by the effect of starch in the burgers with alginate (*p* ≤ 0.05), with the highest values for non-starch samples containing only sodium alginate. This high adhesiveness of alginate is due to its chemical structure, explained before, which allows the formation of ionic bonds that contribute to the adhesiveness of the gel, in addition to its high capacity to retain water in its three-dimensional structure, which allows it to adhere to wet surfaces [41]. In contrast, native starch contains around 25% amylose and 75% amylopectin and is therefore often used as a thickener. However, when starch is subjected to high temperatures during cooking, it loses viscosity and adhesion [13] due to starch gelatinisation, whereby the granules swell and break due to the disruption of the amylopectin double helices by dissociation of the hydrogen bonds, causing a decrease in water holding capacity [42].

Resilience, the property that measures how fast and strong recovery is, showed significant differences because of the hydrocolloid, with alginate showing the highest values for this parameter as compared to methylcellulose (*p* ≤ 0.05), as well as due to the effect of starch in the burgers with methylcellulose (*p* ≤ 0.05), providing greater resilience to those containing starch. This is possibly related to the viscosity of the starch, which was more evident with methylcellulose, as it cannot form ionic bonds as alginate can, which limits its ability to form a strong three-dimensional network, therefore needing starch to provide the samples with greater resilience [13]. Therefore, the higher the ionic content and the number and length of binding sites, the higher the physicochemical properties such as viscosity and gel strength [43]. Hence, burgers with sodium alginate as a binder exhibit a stronger recovery than those with methylcellulose due to their structure [44].

Concerning cohesiveness, an effect of hydrocolloid and starch could also be observed in the alginate samples (*p* ≤ 0.05). These results are consistent with those obtained from cooking losses. Cohesiveness refers to the strength of internal bonds for holding together [45]. The cohesiveness of the alginate was superior to the methylcellulose-containing burgers (*p* ≤ 0.05), although these differences were not very pronounced, as it is the nature of the soy protein that mainly contributes to the three-dimensional internal structure of these emulsions, through hydrophobic interactions and hydrogen and disulphide bonds [46]. Low cohesiveness means that the system-wide formed emulsions are plastic rather than elastic, and this may be a desirable characteristic, as the product would be considered an easy-to-chew food material [45]. In the study by Zhou et al. [36] on meat analogues, results that were very similar to those found in our product were obtained, in terms of cohesiveness and adhesiveness, with the use of soybean as a protein source. However, research by Lee and Hong [7] and Bakhsh et al. [16] on soybean burgers showed lower cohesiveness values than ours, as they described that when the binding agent was increased, the texture profile values increased emulsion proportionally, and the extensive hydration of textured protein with water makes soybean burgers softer and less cohesive.

Mechanical properties are of great importance for meat analogues, as they are responsible for imitating the meaty sensation when consumed [13]. As for the other evaluated parameters, hardness, elasticity, and chewiness, there were no statistically significant differences (*p* > 0.05) for any of the effects we evaluated. Similar results were reported by Peñaranda et al. [23] on textured pea burgers, where no differences were observed in the parameters of hardness, elasticity, or chewiness, with scores similar to those of the present study. In the work by Zhou et al. [36] on commercial textured soybean burgers, the authors obtained similar results to our study, in terms of hardness and cohesiveness, and higher values for resilience, elasticity and chewiness.

It was observed that the source of protein used, the binding agent, and the moisture content in plant-based burgers are factors responsible for their mechanical properties; therefore, they must be taken into account when trying to achieve good textural properties in these products, since decreasing the moisture content and increasing the protein content would result in a more fibrous, cohesive, and elastic structure with lower toughness and chewiness [47].

### 3.2. Sensory Analysis

**Figure 2:**
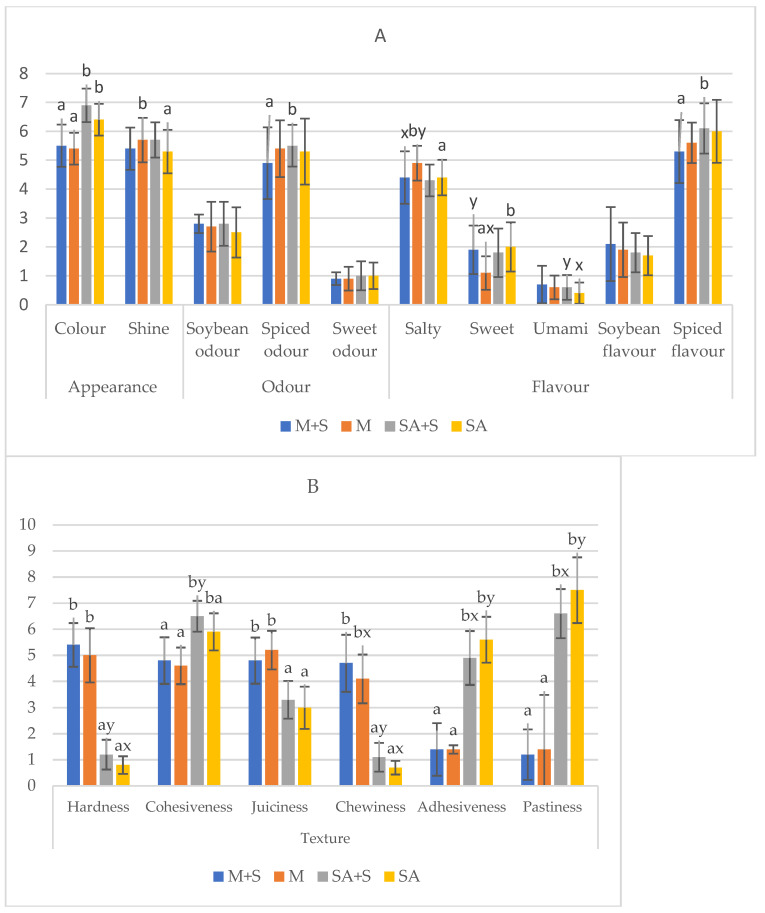
Sensory profile of plant-based burgers with different hydrocolloids: (**A**) appearance, odour, and flavour; (**B**) texture). Mean values ± standard deviations. M+S (burgers made with methylcellulose as a binder with starch), M (burgers made with methylcellulose as a binder without starch), SA+S (burgers made with sodium alginate as a binder with starch), and SA (burgers made with sodium alginate as a binder without starch). Values within a row for formulations with different superscripts significantly differ at *p* ≤ 0.05. ^a, b^: effect of hydrocolloid. ^x, y^: effect of starch. Scores are from 0—not perceptible—to 10—maximally perceptible—on an unstructured 10-point scale.

The results of the sensory analysis of the four formulations of soybean burgers are presented in Table 5 and Figure 2, with statistically significant differences (*p* ≤ 0.05) observed in all the analysed sensory attributes due to the effect of the hydrocolloid used in each formulation (*p* ≤ 0.05), except for the attributes of soybean and sweet odour, umami, and soybean flavour (*p* > 0.05).

As for the colour of the plant-based burgers, it was observed that those containing methylcellulose had a more characteristic colour of soybean, typical of these products. There was a light negative correlation (−0.375 and −0.354, *p* ≤ 0.05) between b* and C* and the colour analysed by the sensory panel. Similar colour scores were obtained in the work of Bakhsh et al. [16] on soybean burgers with different concentrations of methylcellulose.

For shine, the methylcellulose sample without starch obtained the highest values for this attribute, as compared to SA+S (*p* ≤ 0.05). Shine is determined by the amount of water or fat that has migrated to the surface of the food and is capable of reflecting light [48]. Hence, the sample with methylcellulose had the highest shine, as it had a lower water holding capacity than those with alginate.

No significant differences were observed between formulations in the attributes of sweet and soybean odour/flavour, with all samples obtaining scores of around 2.52–2.84 and 0.92–0.96, respectively. For spiced odour, higher scores of around 5 were obtained, with the SA+S samples showing higher scores for this attribute (*p* ≤ 0.05). Spices and herbs with intense odours are normally used in this type of product to mask the unpleasant vegetable or legume connotations characteristic of these products [3,9,23]. Therefore, the volatile compounds responsible for the soybean odour were minimised by the different spices used in the formulations, resulting in the soybean odour and flavour scores being lower than the spice scores [49]. These scores could also be related to the occurrence of Maillard reactions during cooking, which produce aromatic volatiles that could decrease the perception of legume odour and flavour [50].

For the basic taste, the samples with M were the saltiest and therefore less sweet than the SA ones (*p* ≤ 0.05), while for the umami taste, no significant differences were observed between hydrocolloids (*p* > 0.05). Both methylcellulose and sodium alginate are tasteless hydrocolloids, and these flavours tasted are provided by the other ingredients and/or spices added during processing [51]. In general, the use of spices was observed to reduce the characteristic odours and flavours of soybean, which are undesired attributes according to consumers [3], but this combination of spices was not sufficient to grant the final product with a certain umami flavour.

For the texture attributes, it was observed that the plant-based burgers made with methylcellulose were harder, juicier, and chewier and less cohesive, adhesive, and pasty than those made with alginate (*p* ≤ 0.05), presenting scores for hardness, juiciness, and chewiness similar to those reported for meat burgers [41]. For hardness and chewiness, no differences in mechanical properties were obtained. However, the differences perceived between samples by the panellists are probably due to the more pasty and viscous texture of the sodium alginate gel, which may have interfered with these attributes [43]. In addition, for juiciness, it was expected that the plant-based burgers made with sodium alginate would be the juiciest, as it has been found to be related to the ability to retain water by capillarity (WHC and CL) [46]. However, it was found that the juiciest ones were those made with methylcellulose. These values could be influenced by the high pastiness scores given by the panellists to the samples with alginate, as these attributes are opposites; pastiness refers to a thick, viscous, and stickier texture typical of foods that tend to lack juices or liquids, while juiciness refers to the presence of moisture in foods [52]. In this sense, a highly significant and strong negative correlation has been observed (−0.839; *p* ≤ 0.01) between juiciness and pastiness. On the contrary, juiciness had a negative correlation with WHC (−0.632; *p* ≤ 0.01) and a positive correlation with CL (0.467; *p* ≤ 0.01). This shows that the perception of juiciness is not only linked to the amount of water in the product.

For cohesiveness, a similar trend to that shown for mechanical properties was observed, in which the hydrocolloid sodium alginate, due to its ability to form a strong structure [13], provided more cohesive burgers, regardless of whether they were made with added starch or not. Indeed, cohesiveness was positively correlated with WHC (0.532; *p* ≤ 0.01).

As for the effect of starch, significant differences were observed for salty and sweet taste in the samples with methylcellulose, with those without starch being the saltiest and thus the least sweet (*p* ≤ 0.05). Starch itself does not have a sweet taste, but under certain circumstances, such as cooking or mouth digestion, it can release sugars and give rise to a slightly sweet taste in foods, as it is composed of glucose units [42]. As for the umami taste in the samples with alginate, those with added starch in their formulation had the highest values (*p* ≤ 0.05). The umami taste, savoury or delicious, is mainly found in foods such as meat and fish but can be enhanced using seasonings [53]. In our case, the use of spices was not sufficient to intensify this taste and mask the undesirable attributes often associated with proteins of natural origin made from legumes [54]; although differences were observed in general, the scores for this taste in all formulations were practically insignificant at around 0.38–0.69.

In general, the use of starch had a greater effect on texture parameters, where the use of starch provided greater hardness and cohesiveness and less pastiness to the alginate samples as well as greater chewiness to the methylcellulose plant-based burgers (*p* ≤ 0.05).

The amylose and amylopectin chains of starch can trap water and other food components and form bonds with each other via hydrogen bridges, resulting in a strong and stable three-dimensional matrix [42], hence the higher hardness, cohesiveness, and chewiness scores of starch-containing samples. As with the mechanical parameters, the high capacity of alginate gel to retain water in its three-dimensional structure led to greater adhesion to the non-starch samples [37], and hence pastiness, as both attributes are associated with the viscosity of a food [52].

## 4. Conclusions

The use of a unique additive as binder for the elaboration of soybean burgers with methylcellulose or sodium alginate without starch resulted in significant changes in the physical–chemical and sensory properties of the final product. These changes can be attributed to the different characteristics and functions of the binders that were used. Therefore, the alginate burgers, due to their stabilizing, thickening, and gelling properties, provided samples with high water retention capacity and a cohesive and adhesive texture, superior to the samples with methylcellulose, without the need to add starch. In the methylcellulose burgers, starch removal provided the best appearance due to gel transparency and correct textural properties

Sensory evaluation revealed that the replacement of starch with methylcellulose and alginate did not have a pronounced impact on the taste and texture of the textured soybean burgers. Overall, the organoleptic properties indicated more intense burgers with methylcellulose as a binder, as these burgers presented hardness, juiciness, and chewiness scores like those reported for meat burgers. On the contrary, the use of sodium alginate provided more cohesive, adhesive, soft, and pasty burgers.

Therefore, this study highlights the importance of carefully considering the binder that is used, as well as its concentration, when trying to achieve a textured soybean burger with optimal characteristics in terms of texture and flavour. These results are of great importance to the food industry, as they serve to promote the use of a 3g/100g methylcellulose solution as the sole binding agent in soybean burgers, to achieve a product with reduced additives. Furthermore, the combination of other protein sources and additional processing techniques could be explored to improve the olfactory–gustatory sensations of these soybean burgers for commercialization.

## Figures and Tables

**Table 1:** Plant-based burger formulation (w:w, g/g).

Scheme	M+S *	M *	SA+S *	SA *
Hydrated soybean protein ^1^	50	50	50	50
Methylcellulose ^2^	50	50	-	-
Sodium alginate ^3^	-	-	50	50
Starch ^4^	5	-	5	-
Spice mixture ^1, *^	2.5	2.5	2.5	2.5
Olive oil ^1^	9	9	9	9
TOTAL (g)	117	112	117	112
* Spice mixture				
Salt			1.16	
Pepper			0.20	
Garlic Powder			0.57	
Brewer’s Yeast			0.57	
TOTAL			2.5	

* M+S (burgers made with methylcellulose as a binder with starch), M (burgers made with methylcellulose as a binder without starch), SA+S (burgers made with sodium alginate as a binder with starch), and SA (burgers made with sodium alginate as a binder without starch). ^1^ Hacendado, Valencia, Spain. ^2^ Methylcellulose 3 g/100 g solution (E-461, Natural de mezclas, Murcia, Spain). ^3^ Sodium alginate 7 g/100 g solution (E-401, Tradissimo, Barcelona, Spain). ^4^ Maizena, Unilever, Barcelona, Spain.

**Table 2:** Definition of sensory attributes.

	Attributes	Definition	Scale
Appearance	Colour	Similarity of the colour tone of the sample to the characteristic colour of a soybean (light brown). Looking at the colour in the cut surface.	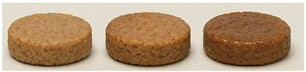 0—light; 5—light brown (characteristic of this product); 10—dark
	Shine	Amount of reflected light on the surface of burger.	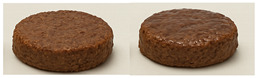 0—dull; 10—very shiny
Odour	Soybean odour	Overall intensity of the soybean or legume odour of the sample.	0—not perceptible; 10—very intense
	Spiced odour	Odour associated with the olfactory perception of a number of spices in the burger.	
	Sweet odour	Odour associated with sucrose.	
Flavour	Salty	Taste sensation associated with the presence of sodium chloride in the food.	0—not perceived; 5—normal salty; 10—very intense
	Sweet	Taste sensation associated with sucrose.	0—not perceived; 10—very intense
	Umami	Taste sensation produced by monosodium glutamate. Induces salivation and a velvety sensation on the tongue.	
	Soybean flavour	Overall intensity of the soybean or legume flavour of the sample.	
	Spiced flavour	Flavour associated with the olfactory–gustatory perception of a number of spices in the burger.	
Texture	Hardness	Force required to deform or compress a substance between the teeth.	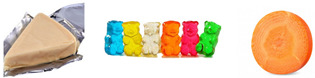 0—easily compressible; 5—characteristic of minced meat; 10—not compressible
	Cohesiveness	Degree of deformation of a foodstuff before it breaks down.	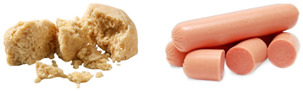 0—little; 10—very much
	Juiciness	Associated with the amount of water and/or fat released from a bite of food during mastication.	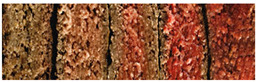 0—completely dry; 10—very moist
	Adhesiveness	Work required by the tongue to dislodge a product stuck on the palate or teeth.	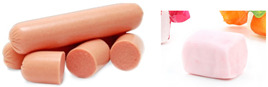 0—little; 10—very much
	Chewiness	Time required to reduce the size of a food until it is swallowed.	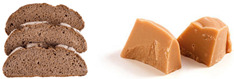 0—no chewing required for swallowing; 10—requires a large amount of chewing for swallowing
	Pastiness	Sensation of paste in the mouth detected. It is an adhesive sample, not very hard and not very elastic.	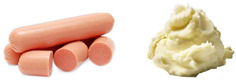 0—little; 10—very much

**Table 3:** Mean values ± standard deviations of colour coordinates, WHC, and CL in plant-based burgers with different hydrocolloids.

CIELab Colour	M+S	M	SA+S	SA
L*	63.55 ± 0.34 ^y^	58.48 ± 0.99^x^	62.31 ± 1.53 ^y^	57.98 ± 0.58 ^x^
a*	7.25 ± 0.17 ^b^	7.68 ± 0.22^b^	6.41 ± 0.26 ^a^	6.78 ± 0.17 ^a^
b*	13.85 ± 0.17 ^x^	14.73 ± 0.31 ^b,y^	12.97 ± 0.41	13.39 ± 0.21 ^a^
C*	15.64 ± 0.21 ^b,x^	16.61 ± 0.37 ^b,y^	14.47 ± 0.47 ^a^	15.02 ± 0.24 ^a^
h*	62.41 ± 0.42	62.52 ± 0.35	63.75 ± 0.50	63.19 ± 0.53
Parameters				
WHC (%)	85.98 ± 0.75 ^a^	84.05 ± 0.66 ^a^	89.21 ± 0.78 ^b^	90.25 ± 1.43 ^b^
CL (%)	14.40 ± 1.70 ^b^	16.52 ± 1.55	9.72 ± 1.20 ^a,x^	14.06 ± 1.26 ^y^

M+S (burgers made with methylcellulose as a binder with starch), M (burgers made with methylcellulose as a binder without starch), SA+S (burgers made with sodium alginate as a binder with starch), and SA (burgers made with sodium alginate as a binder without starch). L*: lightness. a*: red–green. b*: yellow–blue. C*: chroma. h*: ºhue. WHC: water holding capacity. CL: cooking loses. Values within a row for formulations with different superscripts significantly differ at *p* ≤ 0.05. ^a, b^: effect of hydrocolloid. ^x, y^: effect of starch.

**Table 4:** Mean values ± standard deviations of mechanical properties in plant-based burgers with different hydrocolloids.

Properties	M+S	M	SA+S	SA
Hardness 1 (g)	214.0 ± 16.40	251.4 ± 20.10	216.8 ± 23.60	211.9 ± 24.70
Hardness 2 (g)	185.8 ± 12.90	215.6 ± 16.30	193.8 ± 21.10	184.0 ± 20.90
Adhesiveness (mJ)	0.09 ± 0.03	0.19 ± 0.04	0.04 ± 0.03 ^x^	0.21 ± 0.06 ^y^
Resilience (J/m^3^)	0.19 ± 0.01 ^a,y^	0.16 ± 0.01 ^a,x^	0.26 ± 0.02 ^b^	0.22 ± 0.01 ^b^
Cohesiveness	0.53 ± 0.03 ^a^	0.47 ± 0.01 ^a^	0.65 ± 0.03 ^b,y^	0.53 ± 0.00 ^b,x^
Elasticity (mm)	3.29 ± 0.11	3.19 ± 0.07	3.80 ± 0.22	3.33 ± 0.08
Chewiness (mJ)	3.76 ± 0.36	3.74 ± 0.19	5.58 ± 0.95	3.76 ± 0.54

M+S (burgers made with methylcellulose as a binder with starch), M (burgers made with methylcellulose as a binder without starch), SA+S (burgers made with sodium alginate as a binder with starch), and SA (burgers made with sodium alginate as a binder without starch). Values within a row for formulations with different superscripts significantly differ at *p* ≤ 0.05. ^a, b^: effect of hydrocolloid. ^x, y^: effect of starch.

**Table 5:** Sensory profile of plant-based burgers with different hydrocolloids. Mean values ± standard deviations.

	Attributes	M+S	M	SA+S	SA
Appearance	Colour	5.5 ± 0.73 ^a^	5.4 ± 0.55 ^a^	6.9 ± 0.58 ^b^	6.4 ± 0.55 ^b^
	Shine	5.4 ± 0.73	5.7 ± 0.77 ^b^	5.7 ± 0.61	5.3 ± 0.75 ^a^
Odour	Soybean odour	2.8 ± 0.32	2.7 ± 0.86	2.8 ± 0.76	2.5 ± 0.87
	Spiced odour	4.9 ± 1.24 ^a^	5.4 ± 0.98	5.5 ± 0.72 ^b^	5.3 ± 1.14
	Sweet odour	0.9 ± 0.22	0.9 ± 0.41	1.0 ± 0.50	1.0 ± 0.46
Flavour	Salty	4.4 ± 0.91 ^x^	4.9 ± 0.60 ^b,y^	4.3 ± 0.55	4.4 ± 0.61 ^a^
	Sweet	1.9 ± 0.84 ^y^	1.1 ± 0.58 ^a,x^	1.8 ± 0.84	2.0. ± 0.85 ^b^
	Umami	0.7 ± 0.65	0.6 ± 0.41	0.6 ± 0.43 ^y^	0.4 ± 0.37 ^x^
	Soybean flavour	2.1 ± 1.28	1.9 ± 0.94	1.8 ± 0.68	1.7 ± 0.68
	Spiced flavour	5.3 ± 1.09 ^a^	5.6 ± 0.70	6.1 ± 0.87 ^b^	6.0 ± 1.09
Texture	Hardness	5.4 ± 0.84 ^b^	5.0 ± 1.04 ^b^	1.2 ± 0.57 ^a,y^	0.8 ± 0.34 ^a,x^
	Cohesiveness	4.8 ± 0.89 ^a^	4.6 ± 0.70 ^a^	6.5 ± 0.59 ^b,y^	5.9 ± 0.71 ^b,a^
	Juiciness	4.8 ± 0.88 ^b^	5.2 ± 0.74 ^b^	3.3 ± 0.72 ^a^	3.0 ± 0.81 ^a^
	Chewiness	4.7 ± 1.09 ^b,y^	4.1 ± 0.93 ^b,x^	1.1 ± 0.55 ^a,y^	0.7 ± 0.26 ^a,x^
	Adhesiveness	1.4 ± 1.01 ^a^	1.4 ± 0.16 ^a^	4.9 ± 1.03 ^b,x^	5.6 ± 0.88 ^b,y^
	Pastiness	1.2 ± 0.97 ^a^	1.4 ± 2.09 ^a^	6.6 ± 0.94 ^b,x^	7.5 ± 1.26 ^b,y^

Mean values ± standard deviations. M+S (burgers made with methylcellulose as a binder with starch), M (burgers made with methylcellulose as a binder without starch), SA+S (burgers made with sodium alginate as a binder with starch), and SA (burgers made with sodium alginate as a binder without starch). Values within a row for formulations with different superscripts significantly differ at *p* ≤ 0.05. ^a, b^: effect of hydrocolloid. ^x, y^: effect of starch. Scores are from 0—not perceptible—to 10—maximally perceptible—on an unstructured 10-point scale.

## Data Availability

The original contributions presented in the study are included in the article, further inquiries can be directed to the corresponding author.

## Abbreviations

The following abbreviations are used in this manuscript:

WHC	Water holding capacity
M+S	Burgers made with methylcellulose as a binder with starch
M	Burgers made with methylcellulose as a binder without starch
SA+S	Burgers made with sodium alginate as a binder without starch
SA	Burgers made with sodium alginate as a binder without starch
